# Influence of fluid resuscitation on renal microvascular PO_2 _in a normotensive rat model of endotoxemia

**DOI:** 10.1186/cc4948

**Published:** 2006-06-19

**Authors:** Tanja Johannes, Egbert G Mik, Boris Nohé, Nicolaas JH Raat, Klaus E Unertl, Can Ince

**Affiliations:** 1 Department of Physiology, Academic Medical Center, University of Amsterdam, The Netherlands; 2 Department of Anesthesiology and Critical Care, University Hospital Tuebingen, Germany

## Abstract

**Introduction:**

Septic renal failure is often seen in the intensive care unit but its pathogenesis is only partly understood. This study, performed in a normotensive rat model of endotoxemia, tests the hypotheses that endotoxemia impairs renal microvascular PO_2 _(μPO_2_) and oxygen consumption (VO_2,ren_), that endotoxemia is associated with a diminished kidney function, that fluid resuscitation can restore μPO_2_, VO_2,ren _and kidney function, and that colloids are more effective than crystalloids.

**Methods:**

Male Wistar rats received a one-hour intravenous infusion of lipopolysaccharide, followed by resuscitation with HES130/0.4 (Voluven^®^), HES200/0.5 (HES-STERIL^® ^^® ^6%) or Ringer's lactate. The renal μPO_2 _in the cortex and medulla and the renal venous PO_2 _were measured by a recently published phosphorescence lifetime technique.

**Results:**

Endotoxemia induced a reduction in renal blood flow and anuria, while the renal μPO_2 _and VO_2,ren _remained relatively unchanged. Resuscitation restored renal blood flow, renal oxygen delivery and kidney function to baseline values, and was associated with oxygen redistribution showing different patterns for the different compounds used. HES200/0.5 and Ringer's lactate increased the VO_2,ren_, in contrast to HES130/0.4.

**Conclusion:**

The loss of kidney function during endotoxemia could not be explained by an oxygen deficiency. Renal oxygen redistribution could for the first time be demonstrated during fluid resuscitation. HES130/0.4 had no influence on the VO_2,ren _and restored renal function with the least increase in the amount of renal work.

## Introduction

The kidney is one of the most commonly injured organs in critically ill patients. Acute renal failure is a complication in sepsis, with a prevalence ranging from 25% in severe sepsis to 50% in septic shock [1]. Sepsis seems to have an additional impact on outcome, as mortality can be up to 75% among patients with acute septic renal failure [2,3]. The pathogenesis of sepsis-induced renal failure is multifactorial and is characterized by a reduction in the glomerular filtration rate that may occur despite a maintained renal blood flow (RBF) and normal systemic hemodynamics [4].

The morphology of the kidney can range from normal appearing tissue to endothelial damage, medullary blockade with tubular necrosis and disseminated fibrin thrombi [5]. Theories on the pathogenesis suggest an uncontrolled and inappropriate release of various inflammatory mediators leading to direct cytotoxic effects or an impairment of the microvascular autoregulation [6]. The latter might cause a maldistribution of renal microcirculatory blood flow and oxygen supply. Regarding renal tissue oxygenation, there is a high heterogeneity of oxygen tensions within the organ due to the anatomy of the renal microvasculature [7,8]. The fact that not all regions within the kidney are equally well provided with oxygen makes the organ rather sensitive to hypoxic injury [9]. The few studies that have investigated changes in renal tissue oxygenation during endotoxemia present contrasting results [10-12]. The relationship between renal oxygen delivery, consumption and tissue oxygenation, especially with regard to biological response and functional consequences, is still poorly understood and the role of oxygen in septic renal failure remains controversial [10,13,14].

Fluid resuscitation is an early therapeutic strategy in the treatment of septic shock, with the aim of restoring blood flow and oxygen delivery to vital organs [15]. The decision of which solution should be used during resuscitation remains controversial, especially with regard to the kidney. There is an ongoing discussion about the potential of hydroxyethyl starches to impair renal function [16-18]. In well-hydrated patients without preexisting renal dysfunction, however, application of starches seems to be safe [19,20]. Fluid resuscitation not only has an influence on systemic hemodynamics but also dilutes the blood, resulting in beneficial effects on the microvasculature [21,22].

A recently published study from our group demonstrates that resuscitation with HES200/0.5 (HES-STERIL^® ^6%) could successfully restore a decreased mucosal microvascular PO_2 _(μPO_2_) of the pig's intestine after lipopolysaccharide (LPS) infusion [23]. In contrast to the mucosal μPO_2_, the serosal μPO_2 _remained decreased. The gut mucosa and serosa can be regarded as two differently behaving anatomical compartments, and the same accounts for the kidney cortex and the kidney medulla. The renal tissue PO_2 _is regionally different, with values around 50 Torr (6.7 kPa) in the cortex and 20 Torr (2.7 kPa) in the medulla [9]. As the tissue PO_2 _reflects the balance between oxygen delivery and consumption of oxygen in viable cells and tissues [24], its observation in a model of septic renal failure can give important information, particularly because renal hypoxia seems to play an important role in the pathogenesis of the disease [9,25].

The primary objective of the present study is to test the hypothesis that treatment of endotoxemia by fluid resuscitation with either colloids or crystalloids improves an impaired μPO_2_, resulting in restoration of oxygen consumption and kidney function. Secondary to the primary objective our study involves a detailed description of changes in oxygenation during endotoxemia and a comparison of different resuscitation fluids. Four distinct hypotheses can be identified: that renal μPO_2 _and oxygen consumption are impaired during endotoxemia; that this effect is associated with a diminished renal function; that fluid resuscitation with either colloids or crystalloids improves an impaired μPO_2 _and oxygen consumption and restores kidney function; and that colloids are better at resuscitating than crystalloids in this context.

In the present study we applied a new technique recently developed and validated by our group [26] to a normotensive rat model of endotoxemia. This phosphorescence quenching technique allows the noninvasive quantitative measurement of cortical microvascular PO_2 _(cμPO_2_) and medullary microvascular PO_2 _(mμPO_2_) and the detection of the renal venous PO_2 _(P_rv_O_2_). A continuous noninvasive measurement of renal oxygen consumption has been made possible with this unique possibility. Furthermore, we determined the glomerular filtration rate and tubular sodium reabsorption, the major energy-consuming and therefore oxygen-consuming process in the kidney.

## Materials and methods

### Animals

All experiments in this study were approved and reviewed by the Animal Research Committee of the Academic Medical Center at the University of Amsterdam. Care and handling of the animals were in accordance with the guidelines for Institutional and Animal Care and Use Committees. Experiments were performed on 37 Wistar male rats (Charles River, Maastricht, The Netherlands) with a mean ± standard deviation body weight of 282 ± 16 g.

### Surgical preparation

Rats were anesthetized with an intraperitoneal injection of a mixture of 90 mg/kg ketamine (Nimatek^®^; Eurovet, Bladel, The Netherlands), 0.5 mg/kg medetomidine (Domitor^®^; Pfizer, New York, NY, USA) and 0.05 mg/kg atropine-sulfate (Centrafarm, Etten-Leur, The Netherlands). After tracheotomy the animals were mechanically ventilated with a FiO_2 _of 0.4. For drug and fluid administration, four vessels were cannulated with polyethylene catheters (outer diameter, 0.9 mm; Braun, Melsungen, Germany).

A catheter in the right carotid artery was connected to a pressure transducer to monitor the arterial blood pressure and the heart rate. The right jugular vein was cannulated and the catheter tip inserted to a depth close to the right atrium, allowing continuous central venous pressure measurement. Catheters of the same size were placed in the right femoral artery and vein and were used for withdrawal of blood and continuous infusion of Ringer's lactate at a rate of 15 ml/kg/hour (Baxter, Utrecht, The Netherlands). The body temperature of the rat was maintained at 37 ± 0.5°C during the entire experiment. The ventilator settings were adjusted to maintain an arterial PCO_2 _between 35 and 40 Torr (4.7–5.3 kPa). All preceding steps were described in detail in a previous study [27].

The kidney was exposed, decapsulated and immobilized in a Lucite kidney cup (K. Effenberger, Pfaffingen, Germany) via a 4 cm incision of the left flank. The renal vessels were carefully separated from each other under preservation of the nerves. A 0.5 × 1.0 cm^2 ^piece of aluminum foil was placed on the dorsal site of the renal vein to prevent contribution of underlying tissue to the phosphorescence signal in the venous PO_2 _measurement. A perivascular ultrasonic transient time flow probe (type 0.7 RB; Transonic Systems Inc., Ithaca, NY, USA) was placed around the left renal artery and connected to a flow meter (T206; Transonic Systems Inc.) to allow continuous measurement of RBF [28]. The left ureter was isolated, ligated and cannulated with a polyethylene catheter for urine collection. The operation field was covered with plastic foil throughout the entire experiment, to prevent evaporation of body fluids. The experiment was ended by infusion of 1 ml of 3 M potassium chloride inducing a sudden cardiac arrest. Finally, the kidney was removed and weighed, and correct placement of the catheters was checked post mortem.

### Hemodynamic and blood gas measurements

The mean arterial pressure (MAP) was continuously measured in the carotid artery, calculated as: MAP (mmHg) = diastolic pressure + (systolic pressure – diastolic pressure)/3. Furthermore the blood flow of the renal artery (ml/minute) was measured and recorded continuously.

An arterial blood sample (0.2 ml) was taken from the femoral artery at three different time points: first time point, 0 minutes = baseline (*t*_0_); second time point, 50 minutes = endotoxemia (*t*_1_); and third time point, ~70 minutes = resuscitation (*t*_2_). The blood samples were replaced by the same volume of HES130/0.4 (Voluven^®^, 6% HES 130/0.4; Fresenius Kabi Nederland B.V., Schelle, Belgium). The samples were used for determination of blood gas values (ABL505 blood gas analyzer; Radiometer, Copenhagen, Denmark), as well as for determination of the hematocrit concentration, hemoglobin concentration, hemoglobin oxygen saturation, and sodium and potassium concentrations (OSM 3; Radiometer).

### Measurement of renal microvascular oxygenation and renal venous PO_2_

Oxygen-dependent quenching of phosphorescence was used to detect changes in μPO_2 _and to measure the PO_2 _in the renal vein (P_rv_O_2_). In brief, after infusion a water-soluble phosphorescent dye (Oxyphor G2; Oxygen Enterprises, Ltd. Philadelphia, PA, USA) binds to albumin. This phosphor-albumin complex is confined to the circulation and emits phosphorescence with a wavelength around 800 nm, if excited by a flash of light [29]. The phosphorescence intensity decreases at a rate dependent on the surrounding oxygen concentration. The relationship between the measured decay time and the PO_2 _is given by the Stern-Volmer relation: 1/τ = (1/τ_0_) + *k*_*q *_[O_2_], where τ is the measured decay time, τ_0 _is the decay time at an oxygen concentration of zero and *k*_q _is the quenching constant.

For oxygenation measurements within the rat renal cortex and the outer medulla, a dual-wavelength phosphorimeter was used. This new method was recently described and validated elsewhere [26]. Oxyphor G2 (a two-layer glutamate dendrimer of tetra-(4-carboxy-phenyl) benzoporphyrin) gets excited with light of 440 nm and 632 nm, respectively, which allows a continuous and simultaneous measurement in two different depths, the kidney cortex and the outer medulla. On the basis of a high tissue penetration and the fact of the low light absorbance of blood within the near-infrared spectrum, Oxyphor G2 is also well suited for oxygen measurements in full blood. Using a frequency-domain phosphorimeter and a very thin reflection probe, the technique of oxygen-dependent quenching of phosphorescence was applied for noninvasive detection of the P_rv_O_2_.

### Calculation of renal oxygen delivery, renal oxygen consumption, renal oxygen extraction and vascular resistance

Renal oxygen delivery was calculated as DO_2ren _(ml/minute) = RBF × arterial oxygen content (1.31 × hemoglobin × S_a_O_2_) + (0.003 × P_a_O_2_), where S_a_O_2 _is arterial oxygen saturation and P_a_O_2 _is arterial partial pressure of oxygen.

Renal oxygen consumption was calculated as VO_2ren _(ml/minute/g) = RBF × (arterial – renal venous oxygen content difference).

Renal venous oxygen content was calculated as (1.31 × hemoglobin × S_rv_O_2_) + (0.003 × P_rv_O_2_). The S_rv_O_2 _was calculated using Hill's equation with *p*50 = 37 Torr (4.9 kPa) and Hill coefficient = 2.7 [30].

The renal oxygen extraction ratio was calculated as O_2_ER_ren _(%) = VO_2ren_/DO_2ren_.

Since values of renal venous pressure were not available, an estimation of the vascular resistance of the renal artery flow region was made: MAP – RBF ratio (*U*) = (MAP/RBF) × 100 [31].

### Assessment of kidney function

Creatinine clearance (Clear_crea_) was assessed as an index of the glomerular filtration rate according to the standard procedure to measure the function of the investigated kidney [13,32]. Calculations of the clearance were made with the standard formula: clearance (ml/minute) = (*U *× *V*)/*P*, where *U *is the urine concentration of creatinine, *V *is the urine volume per unit time and *P *is the plasma concentration of creatinine. The specific elimination capacity for creatinine of the left kidney was normalized to the organ weight. Urine samples from the left ureter were collected at 10-minute intervals for analysis of urine volume and creatinine concentration. Plasma samples for analysis of creatinine were obtained at the midpoint of each 10-minute urine collection period. The concentrations of creatinine in urine and plasma were determined by colorimetric methods.

Furthermore, all urine samples were analyzed for the sodium concentration. The urine sodium concentration (*U*_Na+_; mmol/l) was multiplied by the urine volume per unit time to obtain sodium excretion (*U*_Na+ _× *V*). The cost of sodium transport (VO_2_/*T*_Na+_) is the ratio of the total amount of VO_2ren _over the total amount of sodium reabsorbed (*T*_Na+_, mmol/minute), which was determined according to: *T*_Na+ _= (Clear_crea _× *P*_Na+_) - *U*_Na+ _× *V*, where *P*_Na+ _is the plasma sodium concentration.

### Experimental protocol

After an operating time of 60 minutes, two optical fibers for phosphorescence measurements were placed both 1 mm above the decapsulated kidney surface and 1 mm above the renal vein. Oxyphor G2 (1.2 ml/kg; Oxygen Enterprises, Ltd) was subsequently infused intravenously for 15 minutes. After 40 minutes μPO_2 _and P_rv_O_2 _were continuously measured during the entire experiment, and 10 minutes later the baseline blood sample (0.2 ml) was taken via the femoral artery catheter. At this time point the rats were randomized between the nonresuscitation group (*n *= 8), the resuscitation with HES130/0.4 group (*n *= 8), the resuscitation with HES200/0.5 group (*n *= 8), the resuscitation with Ringer's lactate group (*n *= 8), and the control group (*n *= 5).

In total 32 animals were assigned to receive a one-hour infusion of LPS (10 mg/kg, serotype 0127:B8; Sigma-Aldrich, Zwijndrecht, the Netherlands) to induce endotoxemia. Five animals served as time controls. A second blood sample was taken 50 minutes after the start of LPS infusion and was analyzed as already described. Directly after cessation of LPS infusion, one group of animals received fluid resuscitation with Voluven^® ^(6% HES 130/0.4; Fresenius Kabi) at a rate of 20 ml/hour. For all resuscitation groups the resuscitation target was defined as a five-minute steady plateau in RBF. A second group of rats received fluid resuscitation with HES200/0.5 (HES-STERIL^®^, 6% HES 200/0.5; Fresenius Kabi) at a rate of 20 ml/hour. A third group of animals received Ringer's lactate (Baxter) as the resuscitation fluid; to ensure the same volume effect, the infusion rate was 60 ml/hour. A fourth group served as controls and did not receive fluid resuscitation after LPS infusion.

The experiment was ended 10 minutes after cessation of fluid resuscitation or at a corresponding time point for the control groups by intravenous bolus injection of 3 M KCl.

### Statistical analysis

Values are reported as the mean ± standard deviation, unless indicated otherwise. The decay curves of phosphorescence were analyzed using Labview 6.1 software (National Instruments, Austin, TX, USA). Statistics were performed using GraphPad Prism version 4.0 for Windows (GraphPad Software, San Diego, CA, USA). Differences within groups were first tested with the one-way analysis of variance for repeated measurements. When appropriate, *post-hoc *analyses were performed with the Student-Newman-Keuls post test. Intergroup differences were analyzed using the unpaired *t *test. *P *< 0.05 was considered significant.

## Results

### Systemic variables

Systemic hemodynamic changes for the time points of baseline (*t*_0_), endotoxemia (*t*_1_) and resuscitation (*t*_2_) are presented in Table 1. Baseline values in the experimental and control groups were no different. LPS infusion induced a slight decrease in the MAP compared with the control group. Resuscitation with HES200/0.5 (HES-STERIL^® ^6%) and Ringer's lactate restored the MAP to baseline values, whereas after resuscitation with HES130/0.4 (Voluven^®^) the MAP remained at 96 ± 26 mmHg. Although the MAP significantly increased in the time control group to 129 ± 6 mmHg at *t*_2_, all groups showed normotensive values during the entire experiment.

After LPS infusion the heart rate increased significantly from 263 ± 21 beats/minute at *t*_0 _to 294 ± 30 beats/minute at *t*_2 _in the nonresuscitation group. The heart rate increased in all groups receiving fluid resuscitation (versus baseline and control values, *P *< 0.05). Fluid resuscitation also increased the central venous pressure significantly regardless of the type of fluid.

The RBF decreased dramatically during LPS infusion to 50% of baseline values and did not recover in the nonresuscitation group. Both resuscitation with colloids and crystalloid restored the RBF to baseline values. After a sudden decrease in RBF from 5.4 ± 1.0 to 2.0 ± 0.7 ml/minute with LPS infusion, HES200/0.5 restored the RBF most effectively to 6.7 ± 1.7 ml/minute (versus baseline and control values, *P *< 0.05).

The calculated renal vascular resistance showed a 50% increase from 26 ± 6 dyne/s/cm^5 ^at baseline to 51 ± 22 dyne/s/cm^5 ^at *t*_2 _in the nonresuscitation group. This increase in renal vascular resistance was present in all groups receiving LPS and could be normalized to baseline values with fluid resuscitation.

The pH remained 7.4 at all time points in the control group. The pH decreased in all groups receiving LPS from 7.4 at baseline, to 7.3 at *t*_1 _and to 7.2 at *t*_2_. Fluid resuscitation could not preserve this drop in pH. The negative base excess decreased from -2.1 ± 2.2 mmol/l for all experimental groups at *t*_0 _to -8.4 ± 2.4 mmol/l at *t*_1_. A further drop to -12.2 ± 6.0 mmol/l in the nonresuscitation group could be prevented by fluid resuscitation.

The resuscitation target for all groups receiving fluid resuscitation was defined as a five-minute steady plateau in RBF. On completion of the experiment, animals resuscitated with HES130/0.4 and HES200/0.5 received an average amount of 5.8 ± 1.3 and 8.0 ± 1.1 ml fluids, respectively, until a plateau in RBF was reached. To reach the same resuscitation target and volume effect, 23.0 ± 4.5 ml Ringer's lactate were administered. Hematocrit values did not change in the control groups. With fluid resuscitation the hematocrit decreased about 21%, 23% and 16% for HES130/0.4, HES200/0.5 and RL, respectively, compared with baseline values.

**Figure 1:**
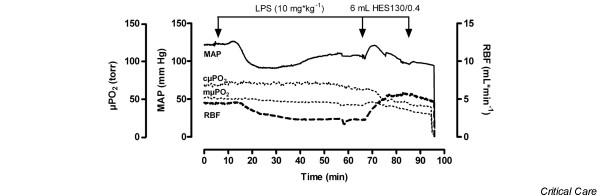
**Example experiment**. Lipopolysaccharide (LPS) infusion resulted in a slight initial decline in the mean arterial pressure (MAP) and a marked decrease in renal blood flow (RBF). Whereas the MAP recovered after 20 minutes, the RBF remained unchanged. Fluid resuscitation with 6 ml HES130/0.4 restored RBF to 20% above baseline values. Cortical (cμPO_2_) and medullary (mμPO_2_) microvascular PO_2 _did not change during LPS infusion. Upon fluid resuscitation cμPO_2 _markedly decreased.

An example of an experiment is shown in Figure 1. The MAP and RBF started to decrease with the onset of LPS infusion. While the MAP dropped only slightly and began to recover to baseline values after 20 minutes, the RBF remained decreased at 50% of baseline. Resuscitation with HES130/0.4 restored RBF to values ~20% above baseline. The cμPO_2 _and the mμPO_2 _only slightly changed during the one-hour LPS infusion. With the onset of fluid resuscitation there was a redistribution of cortical oxygenation towards the medulla.

### Renal oxygenation

**Figure 2:**
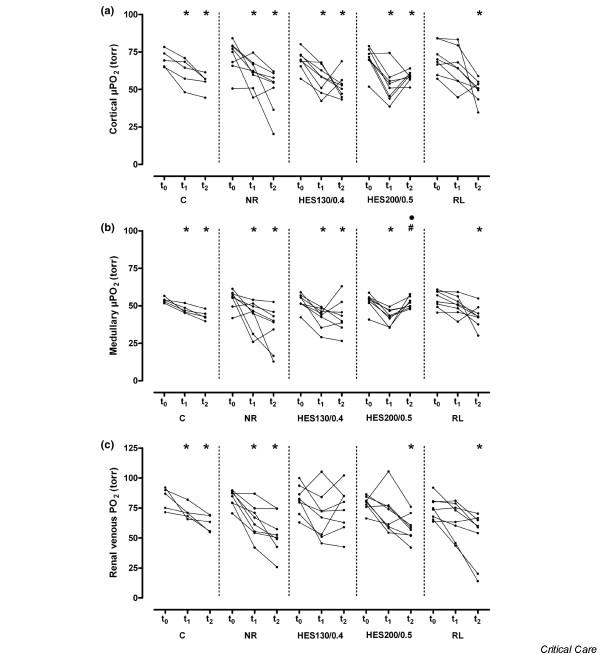
**Measured renal oxygenation parameters**. **(a) **Cortical microvascular PO_2 _(μPO_2_), **(b) **medullary μPO_2 _and **(c) **renal venous PO_2 _at baseline (*t*_0_), endotoxemia (*t*_1_) and resuscitation (*t*_2_) in the control (C) group (*n *= 5), nonresuscitation (NR) group (*n *= 8), HES130/0.4 resuscitation group (*n *= 8), HES200/0.5 resuscitation group (*n *= 8) and Ringer's lactate (RL) resuscitation group (*n *= 8). *P < 0.05 versus baseline, ^#^*P *< 0.05 versus control group, ^•^*P *< 0.05 versus NR group. Rats are individually presented and connected by lines.

**Figure 3:**
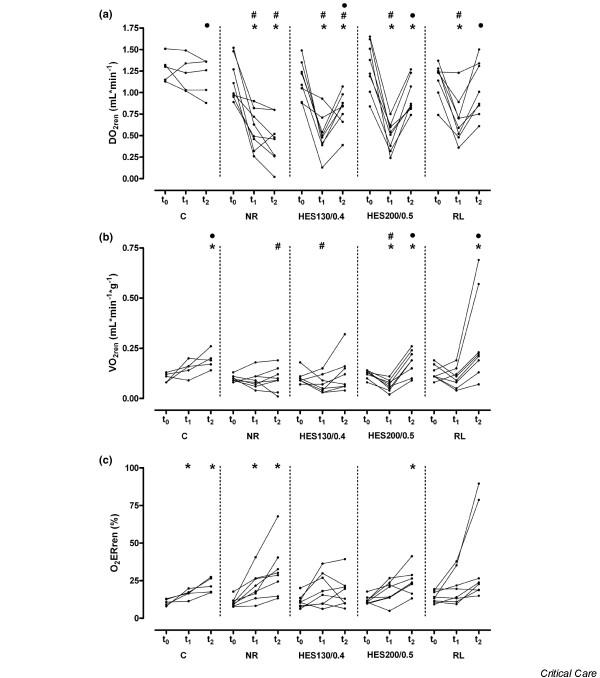
**Calculated renal oxygenation parameters**. **(a) **Renal oxygen delivery (DO_2ren_), **(b) **renal oxygen consumption (VO_2ren_) and **(c) **renal oxygen extraction (O_2_ER_ren_) at baseline (*t*_0_), endotoxemia (*t*_1_) and resuscitation (*t*_2_) in the control (C) group (*n *= 5), nonresuscitation (NR) group (*n *= 8), HES130/0.4 resuscitation group (*n *= 8), HES200/0.5 resuscitation group (*n *= 8) and Ringer's lactate (RL) resuscitation group (*n *= 8). *P < 0.05 versus baseline, ^#^*P *< 0.05 versus control group, ^•^*P *< 0.05 versus NR group. Rats are individually presented and connected by lines.

**Figure 4:**
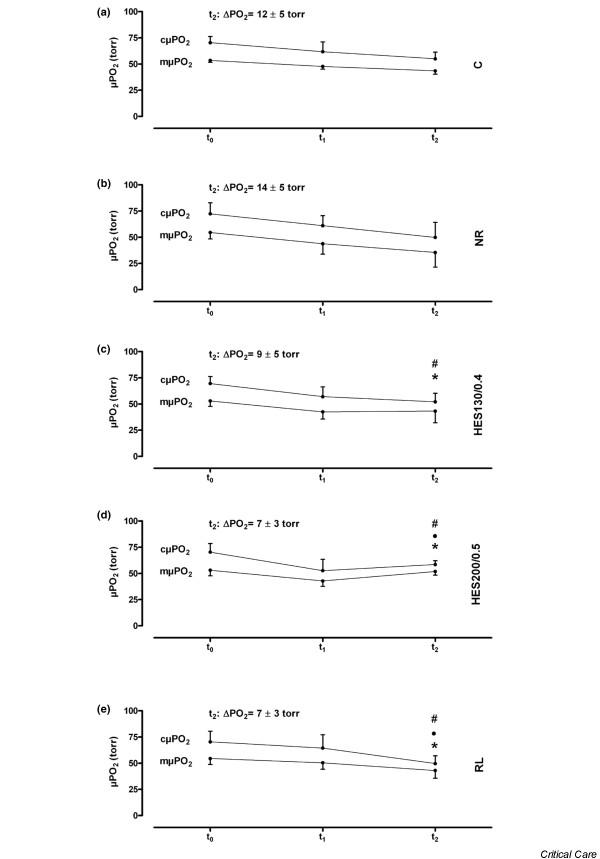
**ΔPO_2 _between cortical and medullary microvascular PO_2 _calculated as a measure of oxygen redistribution**. Cortical microvascular PO_2 _(cμPO_2_) and medullary microvascular PO_2 _(mμPO_2_) are shown for baseline (*t*_0_), endotoxemia (*t*_1_) and resuscitation (*t*_2_) in **(a) **control (C) group (*n *= 5), **(b) **nonresuscitation (NR) group (*n *= 8), **(c) **HES130/0.4 resuscitation group (*n *= 8), **(d) **HES200/0.5 resuscitation group (*n *= 8) and **(e) **Ringer's lactate (RL) resuscitation group (*n *= 8). *P < 0.05 versus baseline, ^#^*P *< 0.05 versus control group, ^•^*P *< 0.05 versus NR group. Data presented as mean ± standard deviation. μPO_2_, microvascular PO_2_. ΔPO_2_, the difference in cμPO_2 _and mμPO_2_.

Data of the oxygenation parameters of the kidney are shown in Figures 2, 3, 4. Baseline values in the experimental and control groups were not significantly different. The cμPO_2 _and mμPO_2 _decreased significantly during the experiment in all groups: from 71 ± 8 Torr (9.5 ± 1.1 kPa) at *t*_0 _to 53 ± 9 Torr (7.1 ± 1.2 kPa) at *t*_2 _for the cμPO_2_, and from 54 ± 5 Torr (7.2 ± 0.7 kPa) at *t*_0 _to 43 ± 10 Torr (5.7 ± 1.3 kPa) at *t*_2 _for the mμPO_2_. LPS infusion had no effect on microvascular oxygenation. The medullary PO_2 _could be significantly restored in animals receiving resuscitation with HES200/0.5 (versus baseline and control values, *P *< 0.05).

The P_rv_O_2 _was significantly lower at *t*_2 _than at baseline in all groups except the HES130/0.4 group. In the group receiving HES130/0.4, the P_rv_O_2 _increased in 50% of the animals and the P_rv_O_2 _was unchanged or decreased in the other 50%, explaining a rather high standard deviation. Although no major changes in renal μPO_2 _occurred, fluid resuscitation regardless of the type of fluid was accompanied by redistribution between the cortical and medullary PO_2_. This redistribution is demonstrated as changes in ΔPO_2 _(shown in Figure 4), defined as the difference in cμPO_2 _and mμPO_2_. cμPO_2 _decreased whereas mμPO_2 _was unchanged in animals receiving HES130/0.4, resulting in a ΔPO_2 _of 9 ± 5 Torr (1.2 ± 0.7 kPa), which was significantly lower compared with baseline and with the control group (ΔPO_2 _12 ± 5 Torr (1.6 ± 0.7 kPa)). In the HES200/0.5 and Ringer's lactate groups, the ΔPO_2 _was 7 ± 3 Torr (0.9 ± 0.4 kPa) respectively. When resuscitated with HES200/0.5 both the cμPO_2 _and mμPO_2 _increased (mμPO_2 _> cμPO_2_), whereas when receiving Ringer's lactate the cμPO_2 _decreased and the mμPO_2 _stayed almost unchanged.

In the experimental groups DO_2ren _decreased immediately during LPS infusion. In the nonresuscitation group DO_2ren _decreased from 1.15 ± 0.25 ml/minute at baseline to 0.58 ± 0.23 ml/minute at *t*_1_, and reached its lowest reading with 0.45 ± 0.27 ml/minute at *t*_2_. Fluid resuscitation restored DO_2ren _to ~0.93 ml/minute, which was slightly but significantly lower than baseline values.

VO_2ren _significantly increased over time from 0.10 ± 0.02 at baseline to 0.18 ± 0.05 ml/minute/g at *t*_2 _in the control group. This increase was not present in animals receiving LPS, in whom VO_2ren _remained around 0.10 ml/minute/g. Fluid resuscitation with HES200/0.5 and Ringer's lactate significantly increased VO_2ren _to 0.18 ± 0.06 ml/minute/g and 0.29 ± 0.22 ml/minute/g, respectively (versus nonresuscitation, *P *< 0.05). Resuscitation with HES130/0.4 had no effect on the renal oxygen consumption. Resuscitation with HES200/0.5 and Ringer's lactate let to a marked increase in O_2_ER_ren _compared with the nonresuscitation group – in contrast to HES130/0.4, which showed no statistical difference.

### Renal function

**Figure 5:**
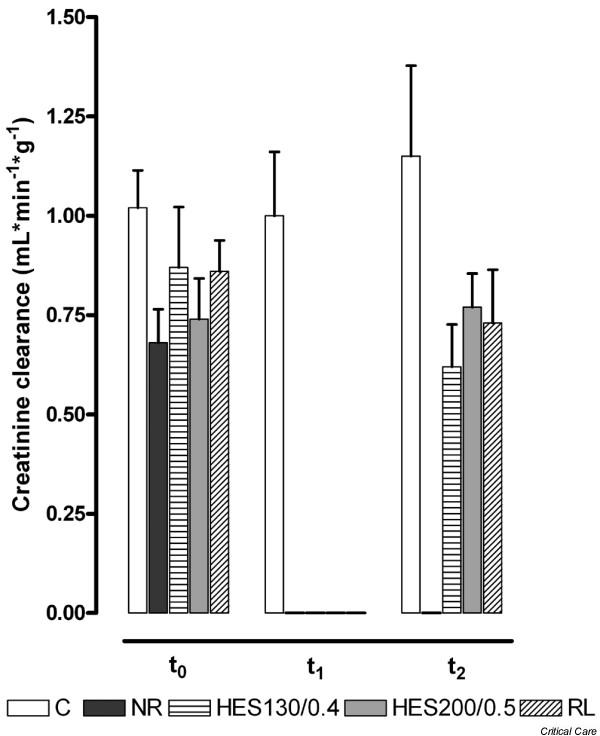
**Creatinine clearance as an index of the glomerular filtration rate**. Creatinine clearance measured at baseline (*t*_0_), endotoxemia (*t*_1_) and resuscitation (*t*_2_) in the control (C) group (*n *= 5), nonresuscitation (NR) group (*n *= 8), HES130/0.4 resuscitation group (*n *= 8), HES200/0.5 resuscitation group (*n *= 8) and Ringer's lactate (RL) resuscitation group (*n *= 6). As lipopolysaccharide infusion was regularly associated with anuria, no clearance could be calculated for these animals. Data were normalized per gram of left kidney weight. Data presented as mean ± standard error of the mean.

The Clear_crea _of the left kidney did not change over the time in control rats. In the experimental groups the averaged Clear_crea _was 0.78 ± 0.31 ml/minute/g left kidney weight. The averaged weight of the left kidney was 1.26 ± 0.10 g. At time point *t*_1 _all animals receiving LPS were anuric. Fluid resuscitation by all tested fluids restored Clear_crea _to baseline values, as presented in Figure 5.

**Figure 6:**
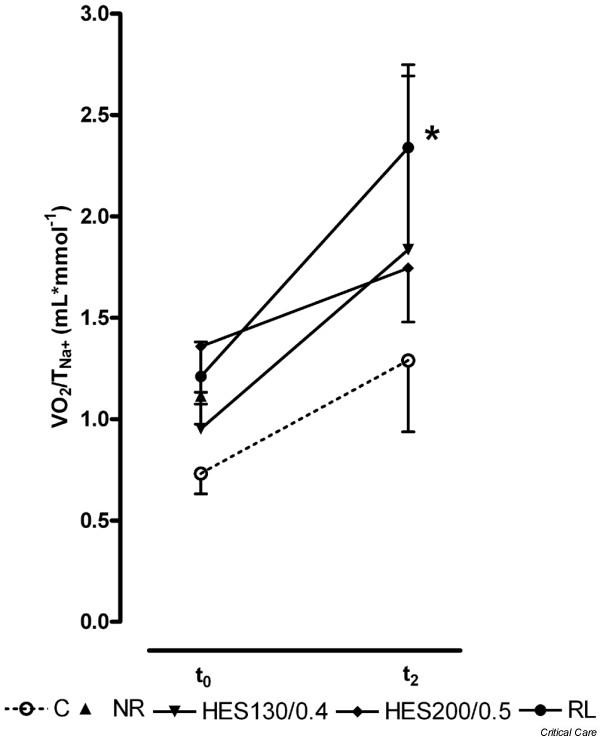
**Kidney oxygen consumption per sodium reabsorbed as an index of metabolic cost**. Oxygen consumption per sodium reabsorbed (VO_2_/T_Na+_) measured at baseline (*t*_0_) and resuscitation (*t*_2_) in the control (C) group (*n *= 5), nonresuscitation (NR) group (*n *= 8), HES130/0.4 resuscitation group (*n *= 8), HES200/0.5 resuscitation group (*n *= 8) and Ringer's lactate (RL) resuscitation group (*n *= 6). *P < 0.05 versus baseline. Testing was performed using the Student paired *t *test. Data are presented as mean ± standard error of the mean.

The baseline values for reabsorptive metabolic costs (VO_2_/*T*_Na+_) were similar in all experimental groups. In the control group the VO_2_/*T*_Na+ _quotient was slightly lower then in the other groups and increased nonsignificantly over the time. With resuscitation VO_2_/*T*_Na+ _trended upwards in all groups. In animals receiving Ringer's lactate the VO_2_/*T*_Na+ _increased from 1.21 ± 0.42 at *t*_0 _to 2.34 ± 0.87 at *t*_2_, which was statistically significant (data shown in Figure 6).

## Discussion

The main findings in our study can be summarized as follows. Endotoxemia severely diminished renal function despite having only a minimal effect on the renal cμPO_2 _and mμPO_2 _and on renal oxygen consumption. Fluid resuscitation restored renal blood flow and re-established kidney function accompanied this by redistribution of μPO_2_. Finally, HES130/0.4 was the only resuscitation fluid tested that did not significantly increase VO_2ren_.

In a normotensive model of endotoxemia we tested four hypotheses: that renal μPO_2 _and renal oxygen consumption are impaired during endotoxemia; that this effect is associated with a diminished kidney function; that treatment of endotoxemia by fluid resuscitation with either colloids or crystalloids can improve an impaired μPO_2 _and oxygen consumption and restore kidney function; and that colloids are more beneficial than crystalloids in this context.

These hypotheses must be partly rejected. As regards the first two hypotheses, in contrast to previous investigation in our model, the renal μPO_2 _and renal oxygen consumption were only minimally affected during endotoxemia, whereas the kidney function was totally diminished. The loss of kidney function therefore cannot be explained by an oxygen deficiency. As hypothesized in the third hypothesis, all resuscitation fluids restored the RBF and kidney function to baseline values. Regardless of which resuscitation fluid was used, oxygen redistribution between the cortex and medulla of the kidney was observed. HES200/0.5 and Ringer's lactate significantly increased the renal oxygen consumption, in contrast to HES130/0.4. Regarding the final hypothesis, as both colloids and crystalloids restored kidney function to baseline values, it might be difficult to choose one in favor of the other. Regarding the renal oxygen consumption, however, renal resuscitation with HES130/0.4 might cause the least amount of renal work.

Administration of LPS was characterized by an increased heart rate, a slight reduction in MAP, a marked decline in RBF, an increase in renal vascular resistance, and a reduction in the glomerular filtration rate resulting in anuria. As the initially slightly reduced MAP recovered to baseline values within 20 minutes, we define our model as normotensive endotoxemia.

This study has some limitations. First, we used anesthetized animals, which could affect the hemodynamic response and renal vascular response to LPS and fluid resuscitation. The changes in the MAP, heart rate and RBF, however, were qualitatively similar to previously published data [13,33,34]. Second, we did not measure lactate or cytokine levels to verify the septic status. We did, however, observe a continuous decrease in blood pH, accompanied by an increase in negative base excess regarded as indicative of sepsis. Third, we used a continuous infusion of LPS to induce endotoxemia. This approach is not the same as human septic shock because our animal model is acute and neglects the effects of disease progression on organ dysfunction over many hours, a problem encountered in many animal models of sepsis [35].

Only a few studies have been performed investigating the behavior of renal tissue oxygenation during endotoxemia. Gullichsen and colleagues [33] observed in dogs that, after endotoxin infusion, the cortical PO_2 _was markedly decreased despite a transient increase in RBF. During the next hours of the experiment the cortical PO_2 _and MAP gradually increased even though the RBF remained relatively depressed. These latter results are in agreement with our observations. Investigations by James and colleagues [11] in mice using electron paramagnetic resonance oximetry showed an initial decrease in cortical PO_2 _and a slight increase in medullary PO_2 _during LPS infusion, which recovered to the control values after 40 minutes. This recovery of PO_2 _in both regions was attributed to a direct toxic effect of LPS on cellular mitochondrial function, resulting in decreased oxygen utilization.

Different findings exist about the influence of endotoxemia on the renal oxygen extraction and oxygen consumption [13,14,33], and the role of oxygen supply herein is controversial. In dogs [33] and sheep [14], for example, an impaired oxygen extraction and decreased oxygen consumption was found. In rats, however, oxygen consumption was not impaired by endotoxemia since the kidney was able to increase the oxygen extraction in the presence of a decreased renal oxygen delivery [13]. An interesting finding in the latter study was that oxygen consumption did not decrease while the effective tubular sodium reabsorption was reduced, indicating that another oxygen-consuming mechanism was induced in their septic model.

Overall, our findings are in agreement with these findings by Heemskerk and colleagues [13]. Oxygen consumption did not change or was only slightly impaired during endotoxemia and increased during resuscitation. The latter increase was accompanied by an increase in the VO_2_/*T*_Na+ _ratio. The causes of that increase in VO_2_/*T*_Na+ _are unknown, but mitochondrial dysfunction (uncoupling) cannot be excluded. In our model μPO_2 _was well preserved during endotoxemia, indicating that although DO_2ren _decreased about 50% this was still sufficient to maintain adequate oxygenation. In our model, therefore, the role of oxygen supply on changes in O_2_ER_ren _and VO_2ren _is likely to be limited. Although no significant difference in baseline oxygenation parameters existed between the groups, it is clear from Figures 2 and 3 that a fairly large variation existed between individual animals in all parameters except the mμPO_2_. The study of Heemskerk and colleagues [13] reported individual values in the same manner and showed similar variations in renal DO_2 _and VO_2 _values.

Several studies indicate an intrarenal redistribution in blood flow from the cortex toward the medulla during endotoxemia [25,36,37]. This suggests that the renal cortex and medulla have different responses to endotoxin in animal experiments. As the renal medulla is especially vulnerable to hypoxia and ischemia [9], sepsis-induced medullary ischemia has been suspected as a major mechanism of such renal injury. In our model the values of cμPO_2 _and mμPO_2 _only declined slightly. The loss in renal function can therefore probably not be attributed to hypoxia. Furthermore, there was no significant evidence of intrarenal redistribution of oxygen during endotoxemia as could be expected with redistribution in blood flow. However, a significant redistribution of oxygen during resuscitation could be observed.

Acute renal failure in sepsis is considered, at least in part, the result of renal ischemia. That is why restoration of adequate RBF by fluid resuscitation should therefore be the primary means of renal protection in critically ill patients. There are few data on the influence of fluid resuscitation on tissue oxygenation [38]. Fluid resuscitation with HES200/0.5 (HES-STERIL^® ^6%) was successful in correcting intestinal mucosal μPO_2_, but not in normalizing serosal μPO_2 _in a normodynamic, low-dose endotoxic pig model [23]. In our model, fluid resuscitation was associated with oxygen redistribution. This redistribution occurred regardless of the type of used fluid and was smallest, although significant, for HES130/0.4. In the HES200/0.5 group both the cμPO_2 _and mμPO_2 _increased, and this may most probably be explained by an increase in cortical and medullary blood flow. When Ringer's lactate was used for resuscitation, the cμPO_2 _decreased whereas the mμPO_2 _was rather unaffected. A possible explanation of the decrease in PO_2 _in the cortex might be due to an increase in oxygen-consuming reabsorption of sodium in the cortical collecting tubules following an increase in the glomerular filtration rate.

In our study all resuscitation fluids were able to normalize the RBF and oxygen delivery. In the only comparable study the gelatin-based resuscitation fluid Haemaccel^® ^was able to restore reduced oxygen delivery during endotoxemia; the oxygen consumption was unaffected [39]. We observed the same effects for HES130/0.4. The application of HES200/0.5 and Ringer's lactate, however, was accompanied by a significant increase in oxygen consumption. Based on the current data we can only speculate on the exact reason(s) why both these fluids significantly increased the renal oxygen consumption compared with baseline values in contrast to HES130/0.4. To reach our chosen resuscitation endpoint (a plateau in RBF) higher volumes of HES200/0.5 and Ringer's lactate where needed, resulting in both a higher volume load and a higher total sodium load. Moreover, systemic hemodynamic parameters were better restored with HES200/0.5 and Ringer's lactate, accompanied by a significant increase in RBF. These factors are all known to influence renal oxygen consumption [40-42]. The increase in renal oxygen consumption was accompanied by an increase in oxygen extraction, and therefore a decrease in renal venous PO_2_, for HES200/0.5 and Ringer's lactate. A point of interest from Figure 6 is that the VO_2_/*T*_Na+ _ratio has the tendency to increase more in the case of HES200/0.5 and Ringer's lactate as compared with HES130/0.4. This indicates that at least part of the oxygen consumption is used by processes other than sodium reabsorption, or alternatively that sodium reabsorption is energetically less effective (higher oxygen cost).

The glomerular filtration rate as an index of kidney function could be restored to baseline values regardless of the type of resuscitation fluid. In two other studies the application of a colloid solution could not restore an endotoxemia-induced reduction in the glomerular filtration rate [32,39], although in our model the fluid resuscitation with the aim of restoring RBF was more aggressive using much higher volumes. The controversy cannot be explained by the fact that we used creatinine clearance as a marker for the glomerular filtration rate/acute renal failure since Heemskerk and colleagues [39] also used creatinine while Heneka and colleagues [32] used inulin clearance. In this respect it is interesting to note that markers for acute renal failure that show a faster response, such as serum cystatin C [43], recently became available and these markers could be the better choice in acute models of kidney failure. It is difficult to arrive at a definitive conclusion regarding the safety of hydroxyethyl starches in respect to renal function. As all fluids restored kidney function during endotoxemia to the same extent, and both crystalloid and colloid solutions increased oxygen consumption during resuscitation, no conclusion can be made in a decision between crystalloids and colloids. HES130/0.4 was able, however, to restore renal function with the least increase in the amount of renal work, as indicated by unaffected renal oxygen consumption. Since under conditions of critical oxygen supply to the tissue one strives to minimize oxygen consumption [44-46], the latter could prove clinically beneficial in more severe shock states.

## Conclusion

The present study for the first time monitored simultaneously and continuously renal cortical and medullary μPO_2 _and renal oxygen consumption in a rat model of endotoxemia and resuscitation. Our model was associated with an impaired kidney function during endotoxemia. As only minor changes in renal μPO_2 _and oxygen consumption could be observed during endotoxemia, the loss of kidney function cannot be explained by an oxygen deficiency. Regardless of the type of resuscitation fluid, the renal blood flow and kidney function could be restored to baseline values. Fluid resuscitation was associated by a redistribution of oxygen, which showed different patterns for the different compounds used. HES200/0.5 and Ringer's lactate increased the renal oxygen consumption, in contrast to HES130/0.4. It can therefore be concluded that fluid resuscitation using HES130/0.4 resuscitates the kidney without increasing the renal work load. This could prove clinically significant in conditions where one strives for oxygen consumption as low as possible.

## Key messages

• 	Endotoxemia severely diminished renal function.

• 	Only minor changes in renal μPO_2 _and VO_2ren _could be observed during endotoxemia, and therefore the loss of kidney function cannot be explained by an oxygen deficiency.

• 	Fluid resuscitation restored RBF and re-established kidney function at the expense of a high VO_2ren_.

• 	HES130/0.4 was the only resuscitation fluid tested that did not significantly increase VO_2ren_.

• 	Fluid resuscitation was accompanied by redistribution between cortical and medullary PO_2._

## Abbreviations

Clear_crea _= creatinine clearance; cμPO_2 _= cortical microvascular PO_2_; DO_2,ren _= renal oxygen delivery; LPS = lipopolysaccharide; MAP = mean arterial pressure; mμPO_2 _= medullary microvascular PO_2_; μPO_2 _= microvascular PO_2_; O_2_ER_ren _= renal oxygen extraction; PO_2 _= partial pressure of oxygen; P_rv_O_2 _= renal venous PO_2_; RBF = renal blood flow; T_Na+ _= tubular sodium reabsorption;VO_2,ren _= renal oxygen consumption;

## Competing interests

The authors declare that they have no competing interests.

## Authors' contributions

TJ made a substantial contribution to the conception and design of the study, performed the animal experiments, was involved in the acquisition, analysis and interpretation of data, and wrote the manuscript. EGM designed and built the technical devices (phosphorimeters), was involved in the interpretation of data and helped to draft the manuscript. BN contributed to the conception and design of study and consulted for clinically relevant importance. NJHR revised the manuscript critically for important intellectual content. KEU gave final approval of the version to be published. CI conceived of the study, participated in coordination and gave final approval of the version to be published. All authors read and approved the final manuscript.

## Figures and Tables

**Table 1:** Systemic hemodynamics

	Baseline (*t*_0_)	Endotoxemia (*t*_1_)	Resuscitation (*t*_2_)
Mean arterial blood pressure (mmHg)			
Nonresuscitation group	117 ± 6	105 ± 13^†^	96 ± 19^*†^
HES130/0.4 (Voluven^®^) group	118 ± 6	102 ± 21^†^	96 ± 26^*†^
HES200/0.5 (HES-STERIL^® ^6%) group	119 ± 9	105 ± 11^†^	114 ± 14
Ringer's lactate group	113 ± 10	102 ± 18^†^	123 ± 26
Control group	113 ± 4	127 ± 3*	129 ± 6^*‡^
Heart rate (beats/minute)			
Nonresuscitation group	263 ± 21	278 ± 27^†^	294 ± 30^*†^
HES130/0.4 (Voluven^®^) group	268 ± 25	277 ± 22^†^	299 ± 24^*†^
HES200/0.5 (HES-STERIL^® ^6%) group	252 ± 16	269 ± 27*	295 ± 16^*†^
Ringer's lactate group	247 ± 14	264 ± 26*	280 ± 19^*†^
Control group	261 ± 9	248 ± 9*	256 ± 7^‡^
Central venous pressure (mmHg)			
Nonresuscitation group	3.8 ± 1.3	3.9 ± 0.6	3.7 ± 0.8
HES130/0.4 (Voluven^®^) group	4.0 ± 0.9	4.0 ± 0.9	6.3 ± 1.2^*†‡^
HES200/0.5 (HES-STERIL^® ^6%) group	4.2 ± 0.6	3.9 ± 0.6	6.0 ± 1.5^*‡^
Ringer's lactate group	3.8 ± 0.9	4.0 ± 1.4	6.7 ± 1.7^*†‡^
Control group	4.2 ± 0.9	4.5 ± 1.8	4.6 ± 1.1
Renal blood flow (ml/minute)			
Nonresuscitation group	4.9 ± 0.9	2.5 ± 1.1^*†^	2.1 ± 1.3^*†^
HES130/0.4 (Voluven^®^) group	4.8 ± 1.0	2.1 ± 0.9^*†^	4.9 ± 1.5^‡^
HES200/0.5 (HES-STERIL^® ^6%) group	5.4 ± 1.0	2.0 ± 0.7^*†^	6.7 ± 1.1^*†‡^
Ringer's lactate group	4.9 ± 0.9	3.0 ± 1.2^*†^	5.7 ± 1.3^*‡^
Control group	5.6 ± 0.9	5.1 ± 1.0	5.1 ± 1.0^‡^
Renal vascular resistance (dyne/s/cm^5^)			
Nonresuscitation group	26 ± 6	50 ± 22^*†^	51 ± 22^*†^
HES130/0.4 (Voluven^®^) group	25 ± 6	58 ± 32*	21 ± 8^‡^
HES200/0.5 (HES-STERIL^® ^6%) group	23 ± 5	57 ± 22^*†^	17 ± 1^†‡^
Ringer's lactate group	24 ± 7	38 ± 9^*†^	21 ± 2^†‡^
Control group	21 ± 3	26 ± 5*	26 ± 5^*‡^

Values presented as the mean ± standard deviation. *P < 0.05 versus baseline, ^†^*P *< 0.05 versus control group, ^‡^*P *< 0.05 versus nonresuscitation group.
